# Role of the mesenchymal stem cells derived from adipose tissue in changing the rate of breast cancer cell proliferation and autophagy, *in vitro* and *in vivo*

**DOI:** 10.22038/ijbms.2020.51461.11678

**Published:** 2021-01

**Authors:** Maryam Adelipour, Abdolamir Allameh, Abdolkarim Sheikhi, Mina Ranjbaran, Mahshid Naghashpour, Zahra Nazeri, Hoda Mojiri-Forushani, Sahar Golabi

**Affiliations:** 1 Department of Clinical Biochemistry, Faculty of Medicine, Ahvaz Jundishapur University of Medical Sciences, Ahvaz, Iran; 2 Department of Clinical Biochemistry, Faculty of Medical Sciences, Tarbiat Modares University, Tehran, Iran; 3 Department of Immunology, Faculty of Medicine, Dezful University of Medical Sciences, Dezful, Iran; 4 Department of Physiology, Faculty of Medicine, Tehran University of Medical Sciences, Tehran, Iran; 5 Abadan Faculty of Medical Science, Abadan, Iran

**Keywords:** Biomarker, Carcinoma, Cell growth, Cell therapy, Molecular pathway

## Abstract

**Objective(s):**

Autophagy is an intracellular degradation system of damaged proteins and organelles; however, the role of autophagy in the progression of cancer remains unclear. In recent years, mesenchymal stem cell (MSC)-based approaches have attracted considerable attention for anti-cancer therapy. The present study aimed to examine the interaction of MSCs with the breast cancer cells under autophagy-induced conditions.

**Materials and Methods:**

In this study, MSCs isolated from human adipose tissue were co-cultured with MDA-MB 231, a breast cancer cell line, and the autophagy process was induced by tunicamycin treatment. The cell viability was monitored by the MTT assay, and the cells were recovered at different time intervals (24 or 48 hours) to determine autophagy markers such as Beclin, mTOR and the ratio of LC3II/I expression. Additionally, the animal study was conducted using a mouse model of breast cancer treated with isogenic adipose-derived MSCs, and the expression of Beclin and Ki67 was determined using immunohistochemistry in breast tumor tissue.

**Results:**

In cancer cells co-cultured with MSCs, the cell proliferation was increased, the Beclin expression and the LC3II/I protein ratio were decreased, and the mTOR expression was increased in MDA-MB 231 upon co-cultured with MSCs. Direct injection of MSCs to a mouse model of breast cancer showed an increase in tumor volume, an increase in the accumulation of Ki67 and a decrease in the Beclin expression in tumor tissues.

**Conclusion:**

The data may suggest that suppressed autophagy in breast cancer cells is probably a mechanism by which MSCs can induce cancer cell proliferation.

## Introduction

According to GLOBCAN, breast cancer has the highest mortality and incidence rates among women in the majority of countries (1). Breast cancer as a heterogeneous cancer has a high degree of diversity among tumors in different patients and even within various tumors of each person (2). 

The current approaches for breast cancer treatment consist of mastectomy and surgery, radiation and systematic therapy such as endocrine therapy, chemotherapy and neoadjuvant and adjuvant therapy. It has been reported that the optimal systematic therapy for breast cancer depends on the expression of estrogen or progesterone receptor and human epidermal growth factor 2 (HER2). Therefore, the treatment of triple negative breast cancer is more complex owing to lack of estrogen, progesterone, and ERBB2 receptors resulting in resistance to targeted therapy (3). 

**Figure 1:**
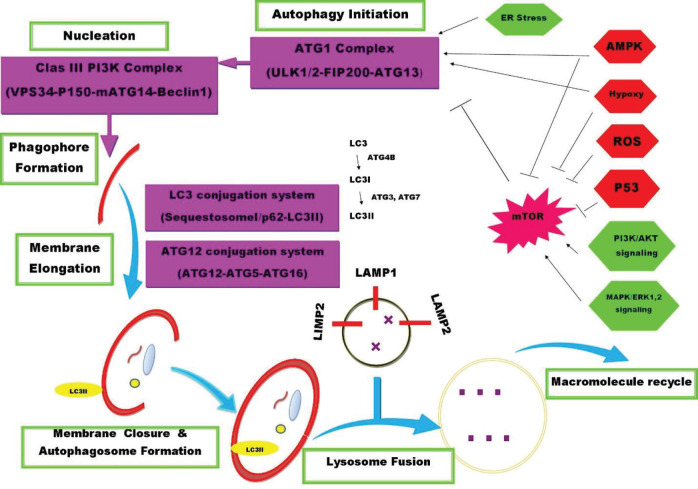
Schematic representation of autophagy signaling. The multistep process of autophagy includes autophagy induction, nucleation, phagophore formation, membrane elongation, membrane closure and autophagosome formation that fuses to the lysosome vacuole for degradation and recycling of macromolecules. Several molecules are involved in the development of these steps, including Autophagy-Related (Atg) Proteins. Autophagy induction occurs under specific conditions such as endoplasmic reticulum (ER) stress, hypoxia and nutrition deprivation. In this condition, Unc-51-like kinase 1 (ULK1) and -2 (ULK2) – two mammalian homologs of yeast Atg1- form a stable complex with the focal adhesion kinase family-interacting protein of 200 kDa (FIP200, homolog of yeast Atg17) and Atg13 that are localized to the phagophore. In this step, the mammalian target of rapamycin (mTOR) as a negative regulator inactivates ULKs and inhibits autophagy. The phagophore nucleation requires the phosphatidylinositol 3-kinase class III (PtdIns3K) complex that consist of PtdIns3K Vps34 (vacuolar protein sorting 34), p150, mAtg14 and Beclin 1. In the next step, cytosolic ubiquitinated substrates are selected and attached to the mammalian protein p62/ sequestosome 1 (SQSTM1). However, p62 is linked to phagophore via LC3 II (microtubule-associated protein 1 light chain 3, the mammalian Atg8 homolog) that is located in the phagophore membrane. In the LC3 conjugation system, LC3I is affected by a cysteine protease named Atg4, conjugated to phosphatidyl ethanolamine and converted to LC3II. LC3 conjugation system, along with another conjugation system consisting of Atg12–Atg5-Atg16 is also necessary for membrane elongation and expansion of autophagosome. Finally, the lysosomal membrane protein lysosome-associated membrane protein 2 (LAMP-2) and the small GTPase Rab7 are required for autophagosome-lysosome fusion (18)

In recent decades, cell-based approaches have attracted considerable attention for their anti-cancer therapy. Mesenchymal stem cells (MSCs) are believed to be good candidates for cell therapy owing to their tumor homing capabilities and lack of immunogenicity (4, 5). Various studies have demonstrated both anti- and pro- tumorigenic effects of MSCs (6-9). Anti-tumorigenic effects of MSCs were indicated by inhibition of angiogenesis and the molecular signaling pathway involved in cell propagation and division. On the contrary, pro-tumorigenic roles of MSCs were shown by suppression of anti-tumor immune response, induction of epithelial-to-mesenchymal transition (EMT), angiogenesis and alteration of tumor metabolic state (4, 10). Therefore, further studies are needed to indicate the ambiguous role of MSCs in targeting the molecular signaling pathway and cancer therapy. Autophagy, as a physiological cellular mechanism involved in the degradation of intracellular damaged proteins and organelles, is a double-edged sword in cancer. Autophagy plays critical roles in both cell death and cell survival depending on the context and stage of tumorigenesis. Autophagy stimulation could be beneficial for cancer prevention during tumor initiation, while it is considered a tumor promoting mechanism by enabling the survival of tumor cells in later stages of cancer (11-13). The autophagy process is divided into several separate steps, including autophagy induction and nucleation, phagophore formation, membrane elongation and phagophore maturation, membrane closure and autophagosome formation and finally autophagsome-vacuole fusion resulting in the breakdown of the cargo and release of degradation products into cytosol (Figure 1). Autophagy induction occurs under specific conditions such as endoplasmic reticulum (ER) stress, hypoxia and nutrition deprivation. Several molecules are involved in the development of these steps that have been usually used as autophagy markers such as Beclin1, the mammalian target of rapamycin (mTOR) and the microtubule-associated protein 1 light chain 3 (LC3)II. Beclin1 is involved in phagophore nucleation; however, mTOR as a negative regulator inactivates Unc-51-like kinase 1 (ULK1) and -2 (ULK2) and inhibits autophagy in the autophagy induction step. In addition, LC3 conjugation system is necessary in the membrane elongation and expansion of the autophagosome step. In the LC3 conjugation system, LC3I is affected by a cysteine protease named Atg4, which is conjugated to phosphatidyl ethanolamine and converted to LC3II. Therefore, an increase in the Beclin and LC3II/I ratio as well as a decrease in mTOR can present activated autophagy in cells (14-18). 

Based on the above information, it was assumed that by changing autophagy in breast cancer cells, in the presence of active MSCs in the system, the effects of these functional stem cells on cancer growth and progression will be more clearly understood. This hypothesis was therefore examined *in vitro* by co-culturing MSCs with the MDA-MB-231breast cancer cell line, and continued with an *in vivo* mouse model of breast cancer.

## Materials and Methods

***Chemicals and reagents***

Dulbecco’s- modified Eagle’s Medium (DMEM), Roswell Park Memorial Institute Medium (RPMI), fetal bovine serum (FBS) and phosphate-buffered saline (PBS) were from Gibco (USA). Penicillin Streptomycin (Pen Strep), Trypsin-EDTA and the MTT (3-(4,5-Dimethyl- 2-thiazolyl)-2,5-diphenyl-2H-tetrazolium bromide) assay Kit were from Bio-idea (Iran). Oil Red O and Alizarin Red S were from Sigma Chemical Company (USA). Anti-CD105, Anti- CD29, Anti-CD90, Anti-CD34 and Anti-CD45 antibodies were obtained from Sigma (USA). LC3B (ab51520), mTOR (ab32028) and Beclin (ab210498) antibodies were from Abcam (USA). The RNA extraction kit was from Gene All (South Korea), and the cDNA synthesis kit was from Solis BioDyne (Estonia). The SYBR Green Master Mix used for real-time PCR was purchased from Ampliqon (Denmark). Tunicamycin was purchased from Sigma–Aldrich (USA) and suspended to distilled water to reach a final concentration of 1 mg/ml.

***Isolation and characterization of MSCs***

In the present study, MSCs derived from human adipose tissue were obtained from Royan Institute. For animal study, adipose tissues were obtained from mouse abdomen during aesthetic surgical procedures and collected in the sterile vessel containing DMEM culture medium. Adipose tissue was rinsed with PBS and digested at 37 °C for 30 min with 0.075% collagenase I to release the cellular fraction. After inactivation of collagenase I with an equal volume of DMEM containing 10% FBS, the infranatant was centrifuged at 500 g for 5 min, and then the pellet was cultured in a culture flask containing DMEM-low glucose supplemented with 10% FBS and 1% streptomycin and incubated at 37 °C in a CO_2_ incubator (5% CO_2_). MSCs were isolated from adipose tissue by their plastic adherent properties. The non-adherent cell fraction was discarded by washing with PBS. The adherent cells were cultured until they reached 70% confluency. The MSCs were then transferred to a new flask and processed for characterization studies. Characterization of the MSCs was routinely examined by identifying their surface markers as well as their differentiation potential concerning adipocytes and osteoblasts. Presence of stemness surface markers, including CD105, CD90 and CD29, and absence of hematopoietic markers such as CD45 and CD34 were determined by flowcytometry (Applied Biosystems, USA). For this purpose, the cells were routinely detached by trypsin/EDTA, and approximately 1× 10^5^ cells were incubated with specific monoclonal antibodies against the mentioned markers or isotype controls in 100 μl of PBS-BSA (3%) for 45 min at 4 °C. 

In addition, the differentiation potential of the MSCs to adipocytes and osteoblasts was examined by adipogenic and osteogenic special culture media in separate preparations. Briefly, differentiation of MSCs to adipocyte was carried out in DMEM enriched with 10% FBS, dexamethasone (10^-6^ M) and ascorbic acid (50 µg/ml) for 2 weeks at 37°C and 5% CO_2_. Then, Oil Red O staining was performed for detection of lipid droplets in adipocytes derived from MSCs. Furthermore, the osteoblast differentiation of MSCs was carried out in DMEM containing 10% FBS, dexamethasone (10^-8^ M), β-glycerol phosphate (10 mM) and ascorbic acid (50 µg/ml) for 3 weeks at 37°C and 5% CO_2_. The mineralized deposits in osteocytes derived from MSCs were detected using Alizarin Red s staining (19).

***Cell culture***

The MDA-MB-231 breast cancer cell line was obtained from Pasteur Institute, Tehran, Iran. The cells were cultured in RPMI medium (GIBCO) supplemented with 10% FBS (GIBCO) and 1% streptomycin at 37 °C, 5 % CO_2_ and humidified atmosphere. The cells were provided with fresh medium twice a week and passaged when they reached 80% confluency.

***Co-Culture system for MSCs and MDA-MB-231 cells***

An indirect cell co-culture using transwell culture dishes (cat numbe#36006, SPL, Korea) was performed to evaluate the interaction of MSCs with the MDA-MB-231 breast cancer cell line. In brief, MDA-MB-231 cells were seeded in a defined medium at 6× 10^5^ cell/well at the bottom of a 6-well dish or 4.52 cm^2^ transwell culture dishes and incubated at 37 °C in a humidified 5% CO_2_ incubator, and allowed to be attached to the plate (3 hr). Then, MSCs were added at a density of 2× 10^5^ on the upper layer of the cell culture dishes and incubated at 37°C for different time periods (24 or 48 hrs). The insert pore size was 0.4 μm as cell migrating was prevented from the inserts to the culture dishes. After 24 or 48 hrs co-culture, the cells at the bottom of culture were used for the MTT assay, as well as RNA and protein extraction.

To examine whether the effects of MSCs derived from adipose tissue on the MDA-MB-231 cells were associated with autophagy pathway, tunicamycin was used as an activator of autophagy to distinguish autophagy induction.

For this purpose, tunicamycin at 2 mg/ml concentration was added to the MDA-MB-231 cell line in the cultured medium before seeded MSCs were derived from adipose tissue on the cell culture insert.

***Cell viability***

Viability of the cells was determined by the MTT assay according to the manufacturing protocol. In brief, MDA-MB-231 cells were cultured (600000 cells/well) overnight in either 6-well tissue culture plates (control) or transwell culture plate (co-culture) containing RPMI supplemented with 2% FBS. After 3 hr, MSCs were seeded at 2× 10^5^ on the cell culture insert of transwell plate and incubated at 37°C for 24 or 48 hr. In addition, tunicamycin (2 mg/ml), as an activator of autophagy for the treatment of MDA-MB-231 cells co-cultured with MSCs, was separately prepared in the cultured medium and incubated at 37°C for 24 or 48 hr. 

After interval times, the medium was changed with fresh serum-free media, and 20 µl of MTT was directly added to the medium to each well with a final concentration of 2 mg/ml. The plates were incubated at 37 °C for 4 hr. The supernatant was then removed, and 300 μl of DMSO was added to each well to solubilize the formazan crystals with shaking for 15 min. Absorbance was measured by a microplate reader at 570 nm, and the viability was expressed as the percentage relative to the untreated control. The experiments were conducted with three samples assayed in triplicate.

***mTOR and Becline expression at mRNA levels***

Expression of mTOR and Becline as autophagy markers was measured in MDA-MB 231 cells, MDA-MB 231 cells co-cultured with MSCs, MDA-MB 231 cells treated with tunicamycin, and MDA-MB 231 cells co-cultured with MSCs treated with tunicamycin by the reverse transcriptase real time PCR (RT-PCR) technique. The specific mRNA from the cells was determined by RT-PCR in the LightCycler 96 Real-Time PCR instrument (Roche, USA). For this purpose, total RNA was extracted from each well using the RNA extraction kit following the manufacturer’s protocol. The synthesis of cDNA was performed using 0.5 μg ofRNA with the FIREScript cDNA synthesis kit, according to the manufacturer’s instruction. Two pairs of specific primers for mTOR, Becline and HPRT (as an endogenous gene) were designed using the CLC software (CLC Genomic Workbench Version 3, 6, 5), and RT-PCR was carried out using the following primers: mTOR Forward, 5’-CCAAAGGCAACAAGCGATCC-3’ and mTOR Reverse, 5’- TGAGAGAAGTCCCGACCAGT -3’; Beclin Forward, 5’- GGAGCTGGAAGACGTGGAAAA-3’ and Beclin Reverse, 5’- AGGTTGCATTAAAGACGTTGG-3’; HPRT Forward, 5’-CCTGGCGTCGTGATTAGTG-3’ and HPRT Reverse, 5’-TCAGTCCTGTCCATAATTAGTCC-3’.

Optimization of the PCR reaction was performed for the primer concentration and the annealing temperature. Gene amplification was performed in 0.2 ml volume microube as the reaction mixture containing 2 µl of diluted cDNA, 5 pmol of each primer, and 5 µl of 2X SYBR green master mixes in a total volume of 10 µl. 

The following PCR program was used; the initial step of 95ºC for 15 min, the amplification step of 40 cycles started with 15 sec at 95ºC followed by 1 min at 60ºC. This program was followed by analyzing the melting curve performed with linear heating from 60 to 90ºC. The fold change of mTOR and Beclin in the cells was calculated according to the following formula: 

Delta Ct = Ct _gene_ – Ct _HPRT_

Delta Delta Ct = ΔCt _treated_ – ΔCt _control_

RQ = Relative Quantitation = 2 ^– (ΔCT treated – ΔCT control)^

All PCR reactions were performed on three samples (n=3) in triplicate. 

***Beclin, mTOR and LC3B expression at protein levels***

Protein expression of Beclin, mTOR and LC3B as autophagy markers was measured in MDA-MB 231 cells and MDA-MB 231 co-cultured with MSCs in the presence or absence of tunicamycin after 48 hr as mentioned in the co-culture design by the western blotting technique. Briefly, the cells were washed with PBS, resuspended in ice-cold lysis buffer and centrifuged at 13000 g for 15 min at 4°C. Supernatants were collected, and protein concentration was determined using the Bradford assay. A total volume of 50 μg protein in loading buffer (25% glycerol, 2% sodium dodecyl sulfate (SDS), 0.01 % bromophenol blue, and 5 % β-mercaptoethanol, pH 6.8) was boiled for 5 min, separated by 12% SDS-PAGE and transferred to a polyvinyldene difluoride (PVDF) membrane. Nonspecific binding of membranes was blocked with 2.5% skim milk and 2.5% glycerol in tris-buffered saline with 0.05% Tween- 20 (TBST). Then, the membranes were incubated with primary antibody (anti-Beclin, anti-mTOR and anti-LC3B antibodies) overnight at 4 °C. After washing with the TBST solution, the membranes were incubated with horseradish peroxidase (HRP)-conjugated secondary antibody (Goat Anti-mouse or Goat Anti-Rabbit (HRP)) for 1 hr at room temperature. The reactive bands of antigen-antibody complexes were detected by a chemiluminescence detection system (Image station 4000MM Pro, Kodak, USA), and western blot bands were measured by the imageJ software.

***In vivo*** **experiment**

In this experiment, 20 inbred female BALB/c 6-week-old mice weighing 15-17 g were purchased from the Pasteur Institute of Iran. The mice were kept under standard conditions with free access to water and food. First, 2 female BALB/c mice were injected with the 4T1 cell line (Cell Bank, Pasteur Institute of Iran) in their left flank region to induce the isogenic experimental model of breast cancer. The tumor was removed after development and used for transplantation to other mice, which were regarded as treated and control groups in the current study. For this purpose, the mice were slightly anesthetized, the subcutaneous pocket was created by blunt dissection in the left flank region, and the fresh tumor piece (1-2 mm) was implanted subcutaneously. Tumor formation was confirmed by showing the growth of the initially grafted tissue 2-3 days after the grafting (10).

Overall, 20 mice bearing breast tumor were divided into two groups that received stem cell or PBS through intra tumor. One week after tumor grafting when the tumor size was 5-7 mm, stem cell therapy was performed. The stem cell-treated groups were injected with MSCs (1×10^6^ cells in 100 µl of PBS/mice) via the intra tumor route. Likewise, the control group was injected only with PBS via the intra tumor route. The tumor diameters were recorded daily, and the tumor volume (V) was calculated using the following formula to quantify the tumor growth:

V (mm^3^)= πab^2^/6 (a= length of tumor, b= width of tumor)

 One month after cell therapy, the mice were sacrificed by cervical dislocation, and tumor biopsies were taken for histopathology studies. Tumor biopsies were fixed in aqueous formaldehyde (10%) before embedding in paraffin blocks and processed for hematoxylin and eosin (H&E) or immunohistochemistry (IHC) staining. In this experiment, Beclin was measured as an autophagy marker, and Ki67 was considered a specific marker for cellular proliferation. Briefly, the blocks were sectioned (4 μm) using microtome and processed for IHC using anti-Beclin and anti-Ki67 primary antibodies (1:100 dilution). Then, the corresponding secondary antibody labeled with HRP was added according to the manufacturer’s instructions. Finally, DAB (3, 3’-diaminobenzidine) and hematoxylin were used as a chromogen to detect antigen and nucleus, respectively. The tissue was observed by a pathologist to grade cancer and determine the presence of focal necrosis. Focal necrosis was calculated by the following scoring number: 0 (without necrosis), +1 (less than 10%), +2 (10%-40%) and +3 (>40%). Additionally, Ki67 and Beclin expression status in sections stained with IHC was estimated as percentage in nuclear and cytoplasm, respectively. 

***Statistical analysis***

Statistical analysis was performed using the SPSS software version 16. The Kolmogorov-Smirnov test was used to find the distribution of data. Differences between the groups were assessed by the one-way ANOVA followed by the Tukey’s test. The significance level was considered at *P*-value≤0.05.

## Results

***Isolation and characterization of MSCs***

**Figure 2:**
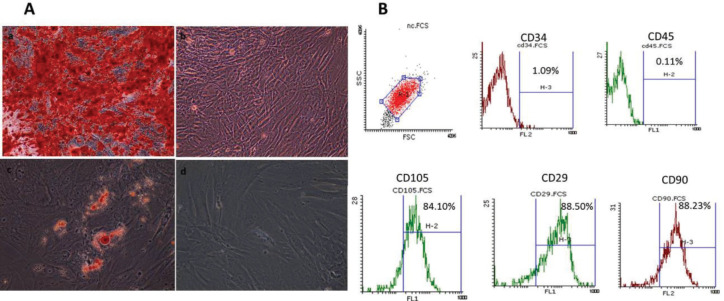
Characterization of MSCs derived from mice adipose tissue. (A) Characterization of mesenchymal stem cells (MSCs) by assessing the differentiation potential of MSCs to adipocytes and osteoblasts. a) Hydroxyapatite crystals synthesized by osteoblasts are detected by Alizarin Red staining. b) Alizarin Red staining of respective control. c) The oil droplets produced in adipocytes are visualized following oil Red staining. d) Oil Red staining of respective control. Original magnification is 200X. (B) Flowcytometric analysis is used to detect cell surface markers. Data show that MSCs express CD105, CD29 and CD90, but they are negative for CD34 and CD45

MSCs derived from adipose tissue were routinely characterized based on the expression of specific CD markers on the cell surface. The results indicated that these cells expressed CD29, CD90 and CD105 markers, whereas they were negative for CD45 and CD34 as shown in Figure 2B. Moreover, as Figure 2A shows, the MSCs derived from adipose tissue could differentiate into osteoblasts and adipocytes in separate setups. The oil droplets produced in adipocytes were visualized following oil Red staining. In addition, hydroxyapatite crystals synthesized by osteoblasts were detected by Alizarin Red staining. 

***The effects of MSCs on MDA-MB231 cell viability***

**Figure 3:**
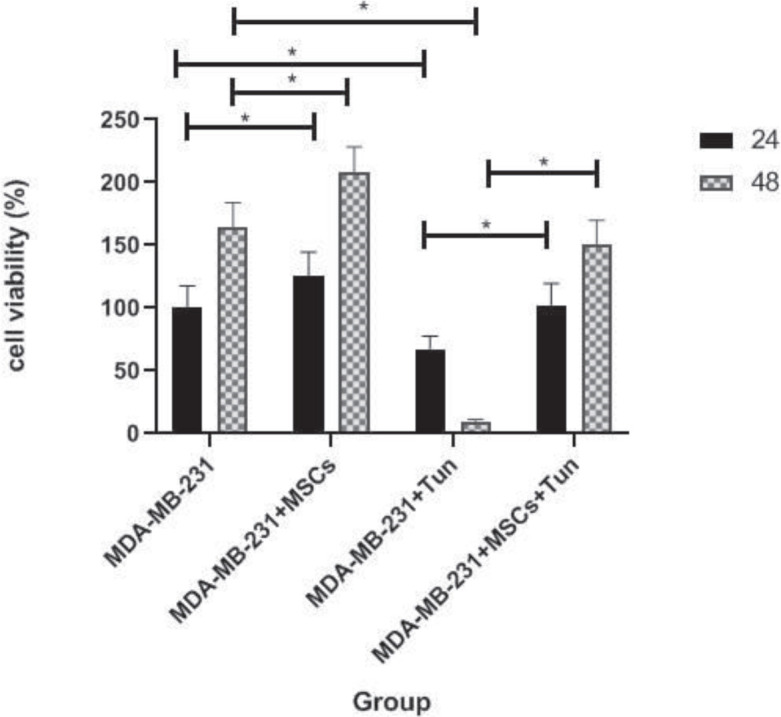
The effect of MSCs, tunicamycin or MSCs along with tunicamycin on breast cancer cell viability. The MTT assay was used to study cell proliferation. Mesenchymal stem cells (MSCs) induce viability of MDA-MB-231 cells in a time-dependent manner. Tunicamycin (2 mg/ml) induces cell death in MDA-MB-231 cells in a time-dependent manner. The viability of MDA-MB 231 cells treated with tunicamycin that were co-cultured with MSCs was increased compared to the viability of MDA-MB 231 cells treated with tunicamycin alone. The viability of untreated cells was set at 100%. Data are the mean+SD of three cell samples performed in triplicate. * *P*<0.05 is considered significantly different between different groups

To evaluate the effects of MSCs derived from adipose tissue on breast carcinoma cell viability, indirect co-culture of the two types of cells was carried out using transwell culture dishes as described in the method section. Then, the viability of MDA-MB 231 cells was determined by the MTT assay. In this assay, tunicamycin was used as an autophagy activator. The results of MTT assays revealed that the viability of MDA-MB231 co-cultured with MSCs derived from adipose tissue was increased significantly compared to untreated MDA-MB 231 as control in a time-dependent manner (~25% in 24 and 48 hr compared to the respective control) (*P*<0.05) (Figure 3). As Figure 3 shows, treatment of MDA-MB 231 cells with tunicamycin resulted in the inhibition of cell viability (~35% and 90% in 24 and 48 hr compared to the control, respectively) (*P*<0.05). Furthermore, the viability of MDA-MB 231 cells treated with tunicamycin that were co-cultured with MSCs derived from adipose tissue were increased compared to the viability of MDA-MB 231 cells treated with tunicamycin alone (~35% and 150% in 24 and 48 hr, respectively) (Figure 3). The data indicated that autophagy induction using tunicamycin can be responsible for the inhibition of cell viability of MDA-MB 231, and co-culturing with MSCs can reverse this effect. 

***Effect of MSCs on expression of mTOR and Beclin in MDA-MB231 cells***

**Figure 4:**
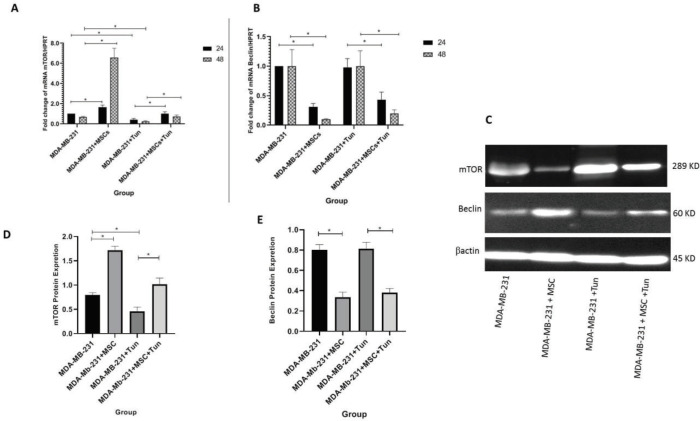
Expression of autophagy markers at mRNA and protein levels in MDA-MB231 cells cultured in the presence of MSCs, tunicamycie or MSCs along with tunicamycin. A) The gene expression of mammalian target of rapamycin (mTOR) in breast cancer cells indicates the upregulation of mTOR in the breast cancer cells co-cultured with mesenchymal stem cells (MSCs), downregulation of mTOR in the cells in the presence of tunicamycin, and upregulation of mTOR in cells co-cultured with MSCs in the presence of tunicamycin compared to tunicamycin alone. B) The gene expression of Beclin in breast cancer cells indicates downregulation of Beclin in cells co-cultured with MSCs. The expression of mTOR or Beclin gene in untreated cells at 24 hr was set at 1 in A, B. C) The expression of Beclin and mTOR at the protein level in MDA-MB-231 cells co-cultured with MSCs. D) The densitometric analysis of the mTOR protein expression in breast cancer cells co-cultured with MSCs after 48 hr. E) The densitometric analysis of the Beclin protein expression in breast cancer cells co-cultured with MSCs after 48 hr. The expression of mTOR or Beclin protein in untreated cells at 48 hr was set at 1 in D,E. Data are the mean+SD of three cell samples performed in triplicate. * *P*<0.05 is considered significantly different between different groups

Comparative expression of mTOR-specific mRNA in MDA-MB 231 cells co-cultured with MSCs derived from adipose tissue using the real-time RT-PCR demonstrated that mTOR was higher in these cells compared to the untreated MDA-MB 231 cells in a time-dependent manner (1.66 and 13 fold at 24 and 48 hr, *P*<0.05) (Figure 4A). The MDA-MB 231 cells treated with tunicamycin as an autophagy activator exhibited a significant decrease in mTOR compared to the respective control (0.42 and 0.36 fold at 24 and 48 hr, *P*<0.05) (Figure 4A). Moreover, the expression of mTOR in MDA-MB 231 cells treated with tunicamycin that were co-cultured with MSCs was increased compared to MDA-MB 231 cells treated with tunicamycin alone (2.38 and 2.9 fold at 24 and 48 hr, *P*<0.05) (Figure 4A). Since mTOR is an inhibitor of the autophagy pathway, the data confirmed that autophagy was decreased in the MDA-MB 231 cells co-cultured in the presence of MSCs derived from the adipose tissue. Therefore, downregulation of mTOR using tunicamycin resulted in autophagy activation in MDA-MB 231 cells.

Moreover, incubation of MDA-MB 231 cells in the presence of MSCs for 24 and 48 hrs resulted in significant downregulation of the Beclin expression (0.31 and 0.1 folds, respectively, *P*<0.05). No changes were observed in the Beclin expression in MDA-MB 231 cells treated with tunicamycin; however, Beclin was downregulated in MDA-MB 231 cells co-cultured with MSCs treated with tunicamycin at 24 and 48 hr compared to MDA-MB 231 cells treated with tunicamycin alone (0.43 and 0.2 folds, respectively, *P*<0.05) (Figure 4B). 

***Protein expression of Beclin, mTOR and LC3B in the MDA-MB231 cells co-cultured with MSCs***

The protein expression of mTOR in MDA-MB 231 cells co-cultured with MSCs (Western blotting technique) showed that the mTOR protein was higher in these cells compared to the untreated MDA-MB 231 cells at 48 hr (2.1 fold, *P*<0.05) (Figure 4C,D). The MDA-MB 231 cells treated with tunicamycin as an autophagy activator exhibited a significant decrease in mTOR compared to the respective control (0.56 fold at 48 hr, *P*<0.05) (Figure 4C, D). The expression of the mTOR protein in MDA-MB 231 cells treated with tunicamycin that were co-cultured with MSCs was increased compared to MDA-MB 231 cells treated with tunicamycin alone (2.2 fold at 48 hr, *P*<0.05) (Figure 4C,D). 

Co-culturing of MDA-MB 231 cells with the MSCs resulted in downregulation of the Beclin protein expression (0.41 fold at 48 hr, *P*<0.05); however no change was found in the Beclin expression in MDA-MB 231 cells treated with tunicamycin (Figure 4C,E). The protein expression of Beclin in MDA-MB 231 cells treated with tunicamycin that were co-cultured with MSCs was downregulated compared to MDA-MB 231 cells treated with tunicamycin alone (0.46 fold at 48 hr, *P*<0.05) (Figure 4C,E).

**Figure 5:**
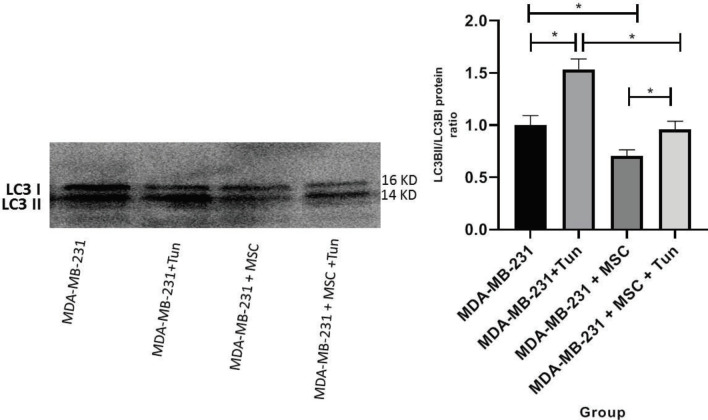
Expression of LC3B I/II protein in MDA-MB231 cells co-cultured with MSCs. The ratio of the LC3BII/LC3BI protein is decreased in breast cancer cells co-cultured with mesenchymal stem cells (MSCs). Tunicamycin induces the change of LC3I to LC3II in MDA-MB231 cells; however, breast cancer cells co-cultured with MSCs treated with tunicamycin showed a decline in the LC3II/LC3I ratio as compared to the cells treated with tunicamycie alone

In addition, we compared the ratio of LC3II/LC3I protein expressions in MDA-MB 231 cells, MDA-MB 231 cell co-cultured with MSCs and MDA-MB 231 cell co-cultured with MSCs treated with tunicamycin. As Figure 5 shows, following the treatment of MDA-MB-231 with MSCs, the ratio of the LC3II/LC3I protein expression was decreased (30% at 48 hr *P*<0.05). Furthermore, MDA-MB231 cells treated with tunicamycin exhibited an increase in the ratio of the LC3II/LC3I protein expression (50% at 48 hr *P*<0.05); however, the ratio of the LC3II/LC3I protein expression was decreased in MDA-MB 231 cell co-cultured with MSCs treated with tunicamycin compared to MDA-MB231 cells treated with tunicamycin alone (~60% at 48 hr *P*<0.05) (Figure 5). Inhibition of autophagy by MSCs suppressed the transformation of LC3I to LC3II, while the elevation of LC3II was shown in the presence of tunicamycin.

***Effect of MSC therapy on tumor growth and histology of breast tumor***

**Figure 6:**
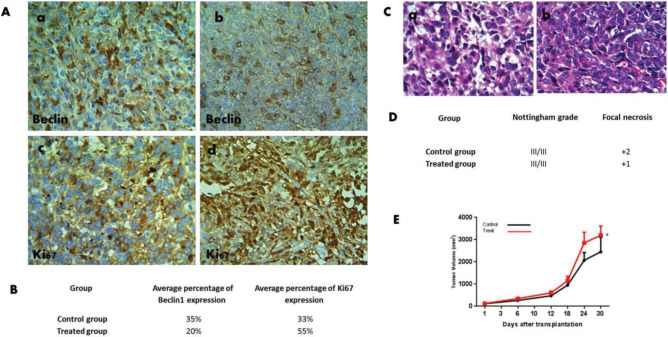
Protein expression of Beclin and Ki67 in tumor tissue of mice treated with MSCs derived from mouse adipose tissue. Section A shows the immunohistochemical analysis of Beclin and Ki67 in the breast tumor of mice treated with mesenchymal stem cells (MSCs) and the control group. Comparison of the Beclin expression in the treated and control groups shown in (a) and (b). (c) and (d) are the breast tumor photographs of mice treated with MSCs and the control group immunostained for Ki67 (Magnification, x400). Section B presents the average percentage of the Beclin and Ki67 expression in the treated and control groups

Comparison of tumor growth in both control and treated groups of mice showed that the MSCs caused a significant increase (*P*<0.05) in tumor volume (Figure 6E). Furthermore, MSCs therapy of breast cancer in the mouse model resulted in changes in breast histology. Histology examination showed the grade III/III for tumor in all of mice with pleomorphism, a high mitotic index and a severe N/C ratio. As Figure 6 C&D shows, the focal necrosis was decreased in the tumors of mice treated with MSCs compared to those in the control group. 

***Expression of Beclin and Ki67 in breast tumor tissues of mice treated with MSCs***

In the animal experiment, Ki67 was considered as specific marker for cellular proliferation. Ki67 in the tumor tissue of mice treated with MSCs was higher than that in the control group as shown in IHC (55% and 33%, respectively) (Figure 6). However, the expression of Beclin as an autophagy marker was lower in mice treated with MSCs than in the control group (20% and 35%, respectively) (Figure 6). The results indicated that autophagy pathway was suppressed in the MSCs-treated group, resulting in cancer cell proliferation.

Section C shows the H&E staining of breast tumor in the control group (a) and the group treated with MSCs (b). Section D presents the results of the histopathological assay in both groups of mice, and section E shows the tumor growth graph of the control and treated groups one month after intervention

**Figure 7:**
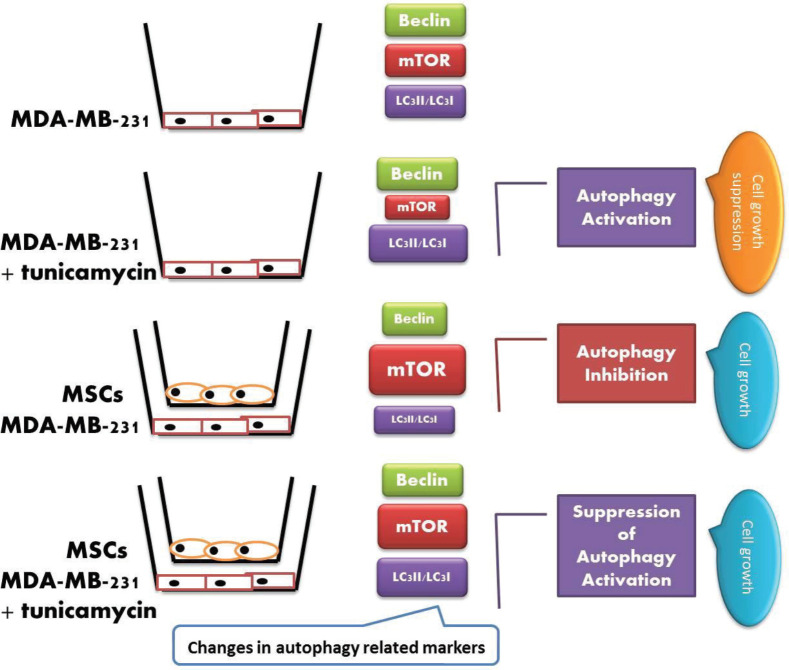
Schematic diagram showing the possible mechanism by which MSCs promote tumor cell growth. Upregulation of mammalian target of rapamycin (mTOR) and downregulation of Beclin-1 and LC3 II in MDA-MB 231 cells treated with mesenchymal stem cells (MSCs) show that MSCs probably act via inhibition of autophagy. Size of the boxes shows the relative rate of the gene or protein expression

## Discussion

Despite the numerous advantages of MSCs in the treatment of various diseases, several challenges concerning the use of MSCs in cancer therapy are raised. Numerous reports have revealed the inhibitory effects of MSCs on the tumor growth (20-22). For example, Li *et al.* demonstrated the inhibitory effect of human adipose tissue MSCs on the growth of lung cancer by mediating the TLR4/NF-kB signaling pathway in mice (23). Furthermore, many studies show the ability of MSCs to promote tumor growth in different ways (24-26). For example, Nishikawa *et al.* showed the secretion of c-c chemokine receptor type5 (CCR5) ligands from bone marrow-derived MSCs that resulted in progression of colorectal cancer (27). Inconsistency in the action of MSCs can result from a different source, a different route of delivery, a different dose/concentration, and different timing of administration (28). In the present study, increased viability of MDA-MB231 cells co-cultured with MSCs derived from adipose tissue compared to MDA-MB 231 cultured alone as control (Figure 3, 7) indicated that bioactive molecules like growth factors derived from MSCs could be probably responsible for the induction of cell proliferation in breast cancer cell line (27). Conditioned media obtained from MSCs cultures contain cytokines and growth factors involved in the cell proliferation process and decreased apoptosis. Hepatocyte growth factor (HGF), Insulin-like growth factor-1 (IGF-1), transforming growth factor β (TGFβ) and basic fibroblast growth factor (bFGF) are among several factors present in conditioned media (29). In this line, Maffey *et al.* showed that MSCs derived from adipose tissue favored breast cancer cell proliferation and metastatic potential via ionotropic purinergic signaling (30).

The evidence indicates that the autophagy process functions either as a tumor suppressor mechanism or as a pro-oncogenic mechanism (31, 32). In this study, it has been demonstrated that autophagy activation in breast cancer cell line (MDA-MB 231) by tunicamycin can lead to decreased cell viability; however, MDA-MB 231 cells co-cultured with MSCs treated with tunicamycin exhibited an increase in cell viability (Figure 3, 6). The preliminary data may suggest that inhibition of autophagy mediators by MSCs can be one of the reasons for an increase in cell proliferation in the breast cancer cell line co-cultured with MSCs. 

Moreover, changes in mTOR and Beclin specific-mRNA or protein expression further confirmed the inhibition of the autophagy pathway in MDA-MB 231 co-cultured with MSCs. Upregulation of mTOR and downregulation of Beclin were indicated in MDA-MB 231 co-cultured with MSCs in both gene and protein expressions. The expression of mTOR and Beclin in the MDA-MB 231 co-cultured with MSCs in the presence of tunicamycin was changed in favor of autophagy activation, along with a decrease in cell growth (Figure 4, 7). Basically, when the Beclin expression is upregulated in the target cells, it can cause the phagophore nucleation step of the autophagy pathway (11).

Changes in the ratio of the LC3II/LC3I protein expression in MDA-MB 231 cells co-cultured with MSCs are other evidences indicating the suppression of autophagy pathways. As a consequence of decline in the LC3II/LC3I ratio, the autophagy may be suppressed in target cancer cells (14, 33). This ratio when reversed in the cells upon treatment with tunicamycin may suggest autophagy activation (34). Inhibition of transformation of LC3I to LC3II in MDA-MB 231 cells co-cultured with MSCs is the good evidence for autophagy inhibition occurred in the cancer cells (Figure 5, 7). Moreover, the LC3II/LC3I ratio was decreased in MDA-MB 231 cells co-cultured with MSCs in the presence of tunicamycin compared to MDA-MB 231 cells treated with tunicamycin alone that suggested the autophagy inhibitory effect of MSCs on breast cancer cells (Figure 5, 7). 

In the present study, intra tumor injection of MSCs resulted in tumor growth compared to the control in the mouse model of breast cancer (Figure 6E). The focal necrosis that is the prominent histopathological observation in breast tumor tissues was inversely associated with tumor growth. Comparison of the histopathological analysis in control and MSC-treated mice suggest that focal necrosis decreases in response to the cell therapy (Figure 6 C&D).

The evidence presented by *in vivo* animal models in cancer or other tissues clearly shows the role of MSCs and their mediators in the suppression of autophagy. The data are converged on the part played by MSCs in changing Beclin and Ki67 factors (35, 36). 

The expression of Beclin in the breast tumor tissue of mice treated with MSCs was downregulated compared to the control group; however, the Ki67 expression was upregulated in the treated group (Figure 6 A&B). Since Beclin is a factor involved in the autophagy pathway, these results confirm the suppression of the autophagy pathway in mice treated with MSCs. 

Considerable evidence indicates modulation of autophagy as an approach in MSCs-based therapy (37, 38). For example, Li *et al.* investigated the protective effects of exosomal miR-301 secreted by bone mesenchymal stem cells (BMSCs) on the autophagy pathway in a model of rats’ myocardial infarction (MI). They showed significantly decreased LC3-II/LC3-I ratio and increased P62 relative expression in infarcted myocardial tissues treated with exosomes carrying overexpressed miR-301, showing myocardial autophagy inhibition (39). Furthermore, according to Song *et al.*, administration of BMSCs significantly ameliorated severe acute pancreatitis-induced multiple-organ injury in rats via suppression of autophagy. They showed that BMSCs acted via inducing PI3K/AKT/mTOR signaling, resulting in inhibition of autophagy. In their study, autophagy inhibition was shown using upregulation of P62 and downregulation of Beclin-1 and LC3 II in pancreatic tissues (36). 

## Conclusion

The data presented in this paper indicate that MSCs, indirectly by their biomolecules, can affect the proliferation rate of MDA-Mb 231 cells. The evidence shows that interaction of MSC with the malignant cells *in vitro* and *in vivo* is associated with changes in autophagy pathways in the cells, which might change the fate of the cancer cells by suppression of autophagy. Further *in vitro* and *in vivo* experiments are needed to understand the mechanism of action of MSC in cancer cells and their biological behavior, particularly their proliferation and apoptosis.
